# Carnosol as a Nrf2 Activator Improves Endothelial Barrier Function Through Antioxidative Mechanisms

**DOI:** 10.3390/ijms20040880

**Published:** 2019-02-18

**Authors:** Xi Li, Qiao Zhang, Ning Hou, Jing Li, Min Liu, Sha Peng, Yuxin Zhang, Yinzhen Luo, Bowen Zhao, Shifeng Wang, Yanling Zhang, Yanjiang Qiao

**Affiliations:** 1 School of Chinese Pharmacy, Beijing University of Chinese Medicine, Beijing 100102, China; xixili1994@163.com (X.L.); zhangqiao0824@foxmail.com (Q.Z.); 13821117290@163.com (N.H.); lijing20171008@163.com (J.L.); liumin_94@163.com (M.L.); moonshadow0406@163.com (S.P.); zhangyuxinwjzy@163.com (Y.Z.); lyz985472@163.com (Y.L.); zhaobw9504@163.com (B.Z.); alana6268@126.com (S.W.); 2 Beijing Key Laboratory of Chinese Materia Medica Foundation and New Drug Research and Development, Beijing 100102, China

**Keywords:** carnosol, antioxidant activity, microvascular endothelial protection, HMVEC cells

## Abstract

Oxidative stress is the main pathogenesis of diabetic microangiopathy, which can cause microvascular endothelial cell damage and destroy vascular barrier. In this study, it is found that carnosol protects human microvascular endothelial cells (HMVEC) through antioxidative mechanisms. First, we measured the antioxidant activity of carnosol. We showed that carnosol pretreatment suppressed tert-butyl hydroperoxide (t-BHP)-induced cell viability, affected the production of lactate dehydrogenase (LDH) as well as reactive oxygen species (ROS), and increased the produce of nitric oxide (NO). Additionally, carnosol promotes the protein expression of vascular endothelial cadherin (VE-cadherin) to keep the integrity of intercellular junctions, which indicated that it protected microvascular barrier in oxidative stress. Meanwhile, we investigated that carnosol can interrupt Nrf2-Keap1 protein−protein interaction and stimulated antioxidant-responsive element (ARE)-driven luciferase activity in vitro. Mechanistically, we showed that carnosol promotes the expression of heme oxygenase 1(HO-1) and nuclear factor-erythroid 2 related factor 2(Nrf2). It can also promote the expression of endothelial nitric oxide synthase (eNOS). Collectively, our data support the notion that carnosol is a protective agent in HMVECs and has the potential for therapeutic use in the treatments of microvascular endothelial cell injury.

## 1. Introduction

Diabetic microangiopathy causes endothelial dysfunction and vascular complications, which is one of the main causes of death and disability in diabetes mellitus [1]. Therefore, it is important for us to study the influence of endothelial dysfunction on diabetic microangiopathy. 

Endothelial cell-to-cell junctions have crucial roles in the process of hyperglycemia vascular endothelial injury because they not only maintain intercellular adhesion, but also transfer intracellular signals that modulate contact inhibition of cell growth, cell polarity, lumen formation, and interactions with pericytes and smooth muscle cells. VE-cadherin is a basic organization of adherens junction protein, which promotes vascular stability, such as protecting cells from apoptosis and controlling the permeability of endothelium [2,3]. Notably, LDH is a stable cytoplasmic enzyme. The leakage of intracellular LDH into the surrounding medium is thought to be the result of cell membrane damage or lysis [4], and this is an irreversible process. Therefore, the increase of LDH can reflect the degree of damage of the cell membrane. In particular, NO can help regulate the normal function of the endothelial barrier and the vascular function eNOS promotes the product of NO in vascular endothelium and blood vessels [5]. 

In 2001, Brownlee [6] proposed the “Common Mechanism of Diabetic Complications” theory that suggests overproduction of superoxide and ROS causes hyperglycemia vascular endothelial injury, which plays a key role in in the mechanisms of diabetic complications. Oxidative stress can significantly increase ROS levels [7,8]. The accumulation of ROS can cause many diseases, including cardiovascular disease, endothelial dysfunction [9] and aging-related diseases [10], as well as neurodegenerative diseases [11]. As a key pathway for oxidative stress, the Keap1/Nrf2/ARE system plays an important role in the study of diabetes [12]. The activation of Nrf2 of human microvascular endothelial cells can significantly improve endothelial dysfunction [13]. When Nrf2 uncouples from Keap1, Keap1 releases Nrf2 and Nrf2 enters the nucleus. Nrf2 can bind its cognate response element, the antioxidant response element (ARE), which upregulates the transcription of ARE-responsive genes, such as HO-1, glutathione (GSH), glutamate-cystein ligase catalytic (GCLC), glutathione-S-transferase (GST) and NADP (H) quinone oxidoreductase 1 (NQO1) [14,15]. 

**Figure 1:**
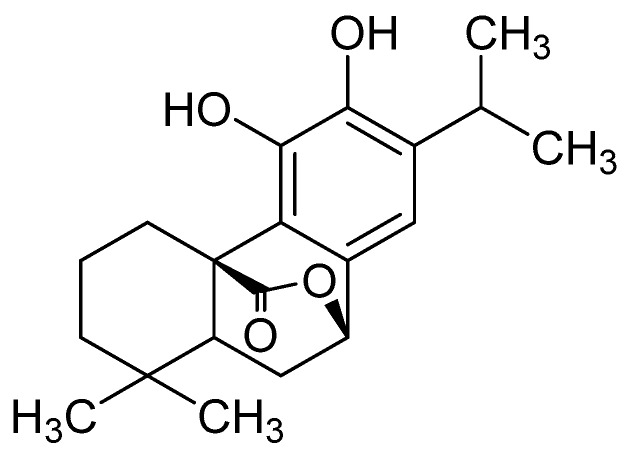
Chemical structure of carnosol.

Natural products play an important role in drug discovery [16]. Rosemary (*Rosmarinus officinalis* L., Lamiaceae) is a woody perennial herb, commonly used for flavoring foods as a condiment [17]. Carnosol (Figure 1) is an anti-inflammatory and anti-oxidant compound which is one of the main components of the extract of rosemary. It has been reported that carnosol also possess potent anti-microbial neuroprotective and anti-tumor properties [18,19,20]. The aim of our study was to investigate the protective effect of carnosol on endothelial damage in HMVEC cells. 

In present research, we first found that carnosol can protect against t-BHP-mediated microvascular endothelial injury in HMVEC cells. Moreover, we evaluated that carnosol can firstly interrupt Nrf2-Keap1 protein−protein interaction. We found that carnosol significantly induces the antioxidant genes and vascular endothelium protection genes upregulation in vitro, such as *HO-1*, *Nrf2* and *eNOS*. For the first time, carnosol was demonstrated that stimulates the VE-cadherin expression in cell-to-cell junctions. We have set up a complete evaluation system which can prove the protective effect of carnosol on microvascular endothelial barrier from phenotype to mechanism in vitro. It has been shown that Tertiary butylhydroquinone (TBHQ) is a positive agonist of Keap1/Nrf2/ARE system and has an effect of improving diabetes mellitus [21]. Therefore, we chose TBHQ as a positive control.

## 2. Results

### 2.1. Carnosol Has the Antioxidant Activity in ABTS Assay

**Figure 2:**
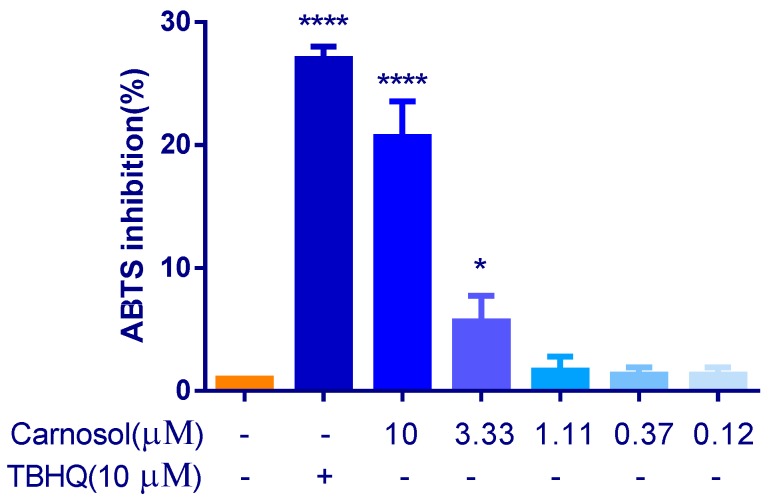
Detection of antioxidant activity of carnosol by ABTS radical scavenging activity. * *p* < 0.05, **** *p* < 0.0001, 0.25% DMSO-treated as negative control group, TBHQ (10 μM) was used as a positive control group, compared with negative group. Results are expressed as mean ± SD (*n* = 3).

2,2′-azino-bis(3-ethylbenzothiazoline-6-sulphonic acid) (ABTS) produces green ABTS^·+^ under the appropriate antioxidants, and ABTS^·+^ is inhibited with the antioxidants. The total antioxidant capacity of the samples can be determined and calculated by measuring the absorbance of ABTS^·+^ at 734 nm. The capacity of carnosol to scavenge ABTS^·+^ was shown in Figure 2, which suggested that carnosol has antioxidant activity. But the antioxidant activity of carnosol was lower than that of 10 μM TBHQ.

### 2.2. Carnosol Can Protect HMVEC Cells Against t-BHP Induced Cell Injury

**Figure 3:**
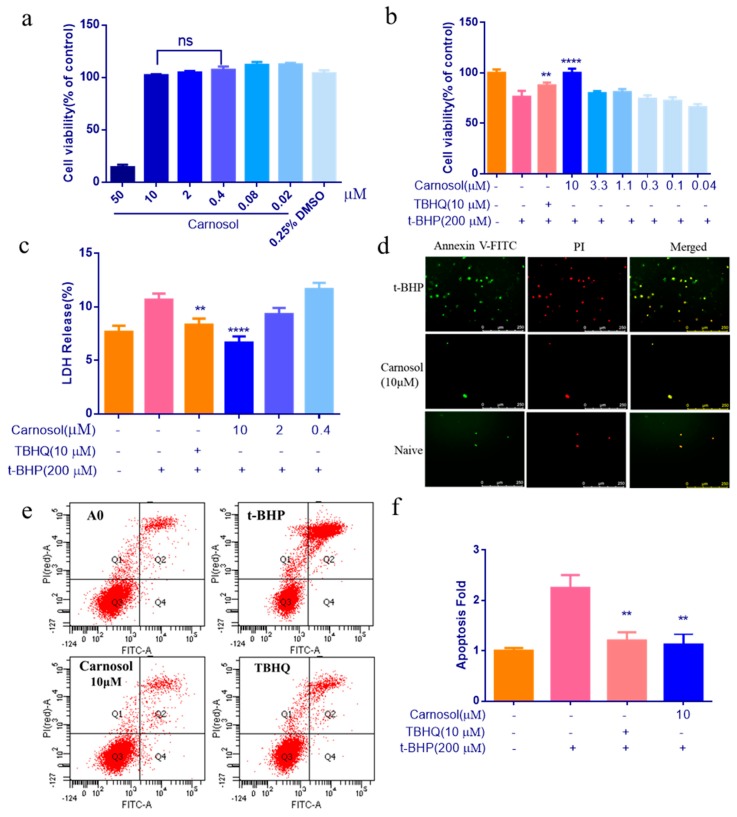
To evaluate the protective effect of carnosol in t-BHP-induced endothelial injury model. (**a**) Evaluating the cell viability of carnosol by CCK-8 assay. (**b**) The cell viability of carnosol pretreated cells after t-BHP-treated for 3 h. (**c**) The levels of the release of LDH were measured using LDH kits. (**d**) the green fluorescence is Annexin V-FITC staining positive cell, and the red fluorescence is propidium iodide (PI) staining positive cell at lower magnification (10×). Apoptotic cells were stained only by green fluorescence, necrotic cells were stained with green and red fluorescence, and normal cells were not stained with fluorescence. (**e**,**f**) Detection of apoptosis by flow cytometry. Apoptotic cells were distributed in Q2 and Q4 regions. ** *p* < 0.01, **** *p* < 0.0001, ns: no significant difference. 200 μM t-BHP-treated as model group, TBHQ (10 μM) was used as a positive control group, 0.25% DMSO-treated as negative control group, compared with model group. Results are expressed as mean ± SD (*n* = 3).

In order to study the effects of carnosol in HMVEC cells, the cytotoxicity of carnosol was first assessed by the CCK-8 assay. The result showed that cells were no cytotoxicity at 10 μM of carnosol (Figure 3a). After treating with 200 μM t-BHP for 3 h, cells have a 20% mortality rate ((Figure 3b). Pretreatment of cells with 10 μM carnosol considerably reduced t-BHP-induced cell injury (Figure 3b). Then, we drew the supernatant to detect LDH. The result suggests that carnosol can significantly reduce the release of LDH (Figure 3c). To evaluate the effect of apoptosis, we used two methods to evaluate apoptosis. After pretreating cells with 10 μM carnosol for 24 h and 200 μM t-BHP for an additional 3 h, we treated with the Annexin V-FITC and PI for 15 min. Then we observed the cell apoptosis of HMVEC cells using fluorescence microscopy. We can obviously observe that 10 μM carnosol can improve cell apoptosis (Figure 3d). Next, we used flow cytometry for a quantitative detection. We found 10 μM carnosol can improve cells apoptosis significantly compared with t-BHP -treated group (Figure 3e,f).

### 2.3. Carnosol Increases the Expression of VE-Cadherin in HMVEC Cells

**Figure 4:**
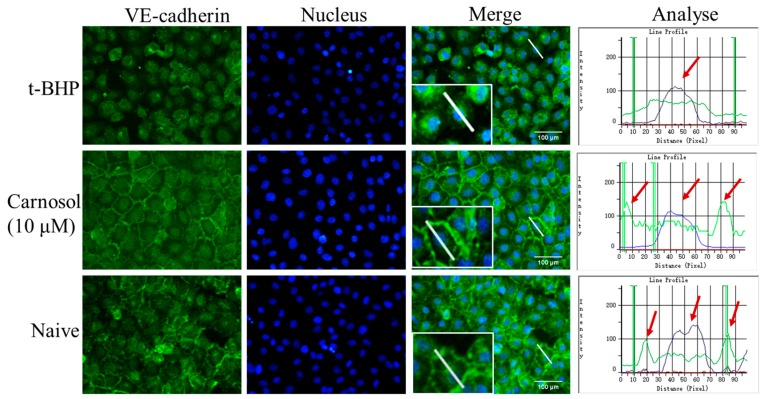
Immunofluorescence staining of VE-cadherin at 20× magnification. VE-cadherin shows a green line on the cell membranes. We continued to enlarge twice in the lower right corner of the merge images. The last row graphs show the relative intensity profiles of fluorescent signal intensities of VE-cadherin (green) and nucleu (blue) along the white line scans depicted in the immunofluorescent images. The *X*-axis indicates the distance of the line and the *Y*-axis represents the fluorescence intensity. The red arrows indicate the location of the peak.

To address the role of carnosol in regulating endothelial barrier function, we first studied the VE-cadherin localization in HMVEC cells. We thus used immunofluorescence to detect the expression and localization of VE-cadherin. After being stimulated with t-BHP 3 h, it disrupted VE-cadherin distribution. In addition, 10 μM carnosol could significantly increase the expression of VE-cadherin. It also maintains the endothelial contacts and adhesion between neighboring cells (Figure 4). The analyzed pictures are the quantification of the expression of VE-cadherin. We measured the fluorescence intensity on a line (white in Figure 4) through the nucleus of the cell in the merge picture. The green line is the fluorescence intensity of VE-cadherin, the blue line is the fluorescence intensity of nucleus, and the peak height indicates the fluorescence intensity. We found that the VE-cadherin intensity of the t-BHP group was reduced. The expression of VE-cadherin was higher in the carnosol group and naive group than in the t-BHP group, and it was shown as a high peak at the junction of cell membrane. 

### 2.4. Carnosol Reduces the ROS Production and Promotes NO Release in t-BHP-Induced Cell Injury in HMVEC Cells

**Figure 5:**
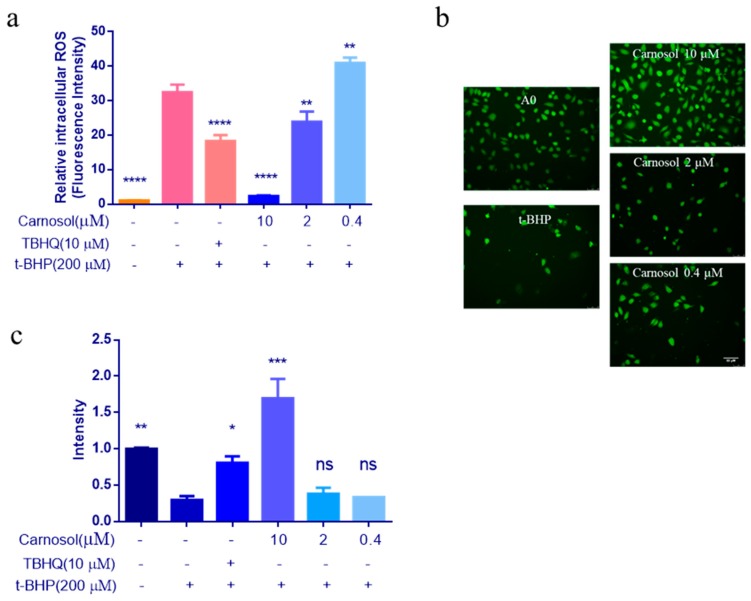
Effects of carnosol in ROS and NO assays. (**a**) The levels of intracellular ROS were measured using DCFH-DA fluorescent probe. (**b**–**c**) The levels of intracellular NO were measured using DAF-FM DA fluorescent probe. It was used a fluorescence microscope to take photos at 20× magnification. * *p* < 0.05, ** *p* < 0.01, *** *p* < 0.001, **** *p* < 0.0001, ns: no significant difference. 200 μM t-BHP-treated as model group, TBHQ (10 μM) was used as a positive control group, 0.25% DMSO-treated as a negative control group, all groups were compared with the model group (no carnosol, no TBHQ, with 200 μM t-BHP).

To confirm the protective properties of carnosol, we investigated whether carnosol countered intracellular ROS and NO production in HMVEC cells treated with 200 μM t-BHP for an additional 3 h. We found ROS production decreased significantly compared with t-BHP -treated group (Figure 5a). Therefore, the results suggested that carnosol might have cytoprotective effects by inhibiting the accumulation of ROS. As shown in Figure 5b, the green fluorescence represents the NO bound by the probe. We can observe that carnosol can significantly promote the release of NO (Figure 5c). 

### 2.5. Carnosol Has the Potential of Interrupting Nrf2-Keap1 Protein−Protein Interaction and Stimulating ARE-Mediated Luciferase Activity

**Figure 6:**
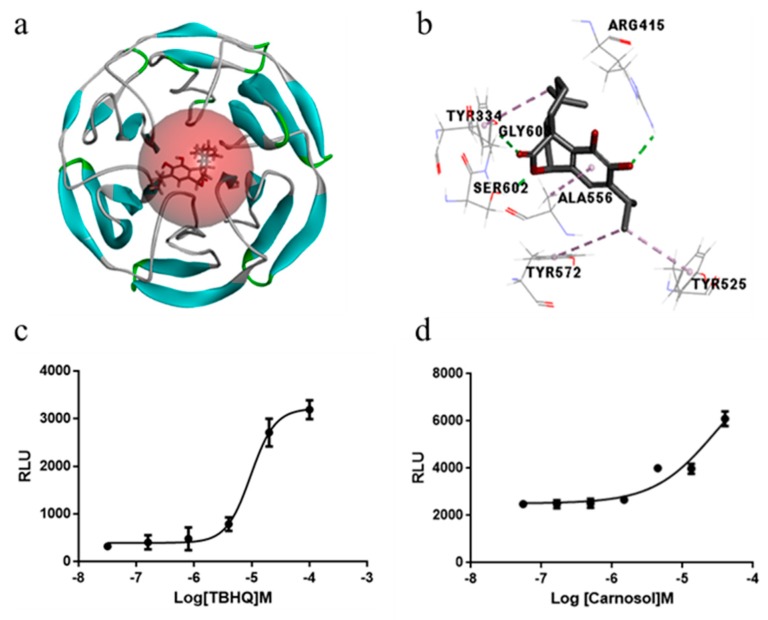
Docking mode of carnosol in the binding site of Keap1 and ARE-mediated luciferase activity. (**a**) Top view of the docking mode of carnosol in the binding site of Keap1. (**b**) The potential interaction between carnosol and Keap1 is via hydrogen bonds and hydrophobic. Potential hydrogen bonds were depicted in green dot lines and hydrophobic were depicted in purple dot lines. (**c**) Dose-response curves of ARE agonist (t-BHP) served as a positive control in this study. (**d**) Carnosol induced ARE-luciferase activity in a dose-dependent manner. Results are expressed as mean ± SD (*n* = 3).

We used molecular docking techniques to analyze whether carnosol can directly inhibit the Nrf2-Keap1 PPI. We found carnosol formed hydrogen bond interactions with amino acids ARG415, SER602, GLY603 and hydrophobic interaction with TRY334, TRY525, TRY572, ALA556(Figure 6a,b). In particular, the interaction pattern was similar to the original ligand, indicating that carnosol has a high potential inhibitory activity. In luciferase reporter activity assay, the dose-response of t-BHP indicates that our cell lines have been successfully constructed (Figure 6c). Then, we proved that carnosol significantly increased ARE luciferase activity in a dose-dependent manner (Figure 6d). 

### 2.6. Carnosol Stimulates the Expression of Cytoprotective Genes and Proteins.

**Figure 7:**
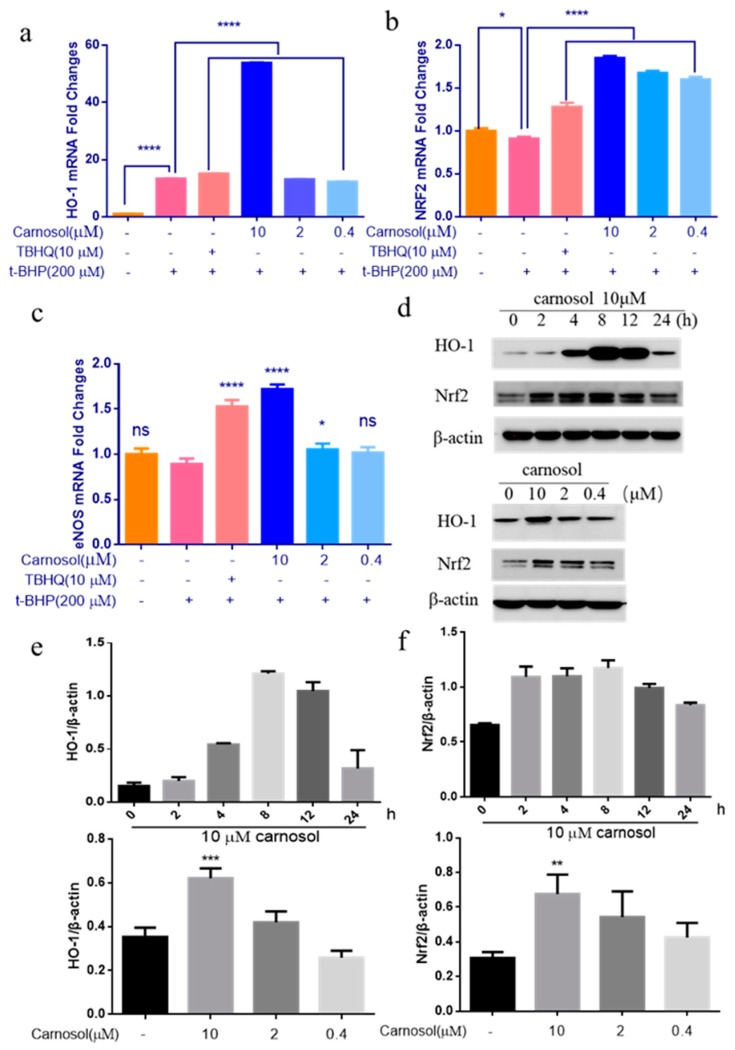
Effects of carnosol on cytoprotective gene and protein expression in HMVEC cells. (**a**–**c**) The mRNA levels of cytoprotective genes, such as *HO-1, Nrf2* and *eNOS* were evaluated using real-time RT-PCR, with GAPDH as an internal control. (**d**) HMVEC cells were treated with 10 μM carnosol for 0, 2, 4, 8, 12 and 24 h (top) or with various concentrations of t-CA for 24 h (bottom). The protein levels of HO-1 and Nrf2 were detected with immunoblotting. (**e**) Levels of HO-1 expression with 10 μM carnosol for 0, 2, 4, 8, 12 and 24 h (top). Levels of HO-1 expression with various concentrations of t-CA for 24 h(bottom). (**f**) Levels of Nrf2 expression with 10 μM carnosol for 0, 2, 4, 8, 12 and 24 h (top). Levels of Nrf2 expression with various concentrations of t-CA for 24 h (bottom). * *p* < 0.05, ** *p* < 0.01, *** *p* < 0.001, **** *p* < 0.0001, 200 μM t-BHP-treated as model group, TBHQ (10 μM) was used as a positive control group, 0.25% DMSO-treated as a negative control group, compared with the model group. Results are expressed as mean ± SD (*n* = 3).

RT-PCR analysis of carnosol-treated cells showed a significant elevation of *eNOS*, *HO-1* and *Nrf2* genes in HMVEC cells. The mRNA levels of increased respectively compared with the control (no carnosol, no TBHQ, with 200 μM t-BHP). These results suggest that 10 μM carnosol treatment could up-regulate *Nrf2*, *HO-1* and *eNOS* genes expression in HMVEC cells (Figure 7a–c). We have also detected the protein levels of HO-1 and Nrf2 in response to the carnosol treatment for 24 h under the doses (Figure 7d). HO-1 and Nrf2-mediated cytoprotective proteins were activated by carnosol at 10 μM. Next, the time dependent inductions of HO-1 and Nrf2 by carnosol was investigated. The protein level of HO-1 in HMVEC cells treated by carnosol increased as early as 4 h, reached the highest level at 8 h, and then gradually decreased (Figure 7e). The protein level of Nrf2 in HMVEC cells treated by carnosol increased as early as 2 h, reached the highest level at 8 h, and then gradually decreased (Figure 7f).

## 3. Discussion

Many natural compounds and natural product mimics are potential antioxidants which can protect against oxidative damage in chronic diseases [22]. Phenolic acids are an important component of natural antioxidants. Carnosol is a kind of diterpenoid, which was known to possess a range of therapeutic effects such as anti-cancer, anti-inflammatory, and anti-oxidant activities in previous studies [23]. Diabetic microangiopathy includes diabetic retinopathy, diabetic nephropathy, and diabetic neuropathy. Most of these diseases interact with endothelial injury and oxidative stress [24,25,26]. Here, we showed that carnosol improves t-BHP-induced endothelial injury in HMVEC Cells.

It has been shown that t-BHP can cause endothelial cell injury through different pathways [27,28]. To establish the model for endothelial injury, we used t-BHP to induce cell injury in this study. LDH is a key marker of cell membrane integrity and cell viability. It is a cytoplasm containing enzymes of living cells. The increase of LDH is usually regarded as a marker of cell disintegration and necrosis [29,30]. We proved that carnosol pretreatment increased cell viability and reduced LDH release caused by t-BHP in HMVEC cells. Abnormal apoptosis may lead to pathological conditions [31]. Apoptosis is an important factor in endothelial protection, and the development of it is directly related to the degree of endothelial damage. We found that carnosol can improve cells apoptosis significantly compared with t-BHP -treated group of seriously endothelial damage. Therefore, we believe that carnosol is a potential drug for endothelial protection.

Disrupting the endothelial cell junctions not only increases vascular permeability, but also changes the response of endothelial cells to their environment and surrounding cells. For instance, VE-cadherin is an important component for the vascular endothelial adhesion and protects the endothelial barrier [3]. Our results show that carnosol can significantly protect the structure of cells by promoting the expression of VE-cadherin. Additionally, the function and organization of VE-cadherin can be regulated by numerous vasoactive agents such as histamine, prostaglandins, thrombin and NO [32]. eNOS produces NO in the blood vessels, while NO helps play crucial roles in regulating vascular function, endothelium inflammatory reaction, as well as vascular growth and regeneration [5]. Carnosol can significantly promote the release of NO and promote the expression of eNOS in HMVEC cells. This may be an important mechanism for enabling carnosol to protect the endothelium barrier.

Oxidative stress is a main cause of the development of diabetic microangiopathy. However, the clinical strategies for the treatment of diabetic microangiopathy by enhancing antioxidant defense mechanisms have not been fully explored. Overproduction ROS production that exceeds the antioxidant capacity leads to cellular oxidative stress and is implicated in a broad range of diseases, such as endothelial injury and cancer. [33]. In particular, ROS contribute to the promotion of the oxidative stress response and the activation Nrf2 signaling [34]. We have demonstrated that carnosol can significantly reduce the production of ROS in HMVEC cells. Keap1/Nrf2/ARE pathway plays an important role in the mediation of health recovery, oxidative stress, diabetes and its complications [35,36]. Nrf2-Keap1 protein-protein interaction (PPI) has been recognized as a key point for regulating Nrf2 activation [37]. Keap1 depends on the interaction with the key tyrosines, namely Tyr525, Tyr574, Arg-415 and Tyr334 [38,39]. We confirmed that Arg415, Tyr334, Tyr525 were key amino acids for the interaction of carnosol with Keap1. Moreover, carnosol can activate the HEK293-ARE cell line with a gradient. Therefore, we considered that carnosol has the potential to activate the Nrf2 and interrupt Nrf2-Keap1 protein−protein interaction. The virtual method also provided a promising strategy to discover Nrf2 inducers. But we still need to prove the effect of carnosol on keap1 protein in our future experiments. Nowadays, Nrf2 is called the "main regulator" of antioxidant reaction. It stimulates the expression of hundreds of genes, most of which encode antioxidant/detoxifying enzymes, such as HO-1 [40]. Indeed, HO-1 is an important enzyme against oxidative damage [41]. So, we focused on the capability of carnosol on up-regulating HO-1 and Nrf2 gene and protein expression in HMVEC cells. 

In summary, this study revealed that carnosol strongly induced Nrf2 activation in HMVEC cells and prevented endothelial injury. Carnosol protects HMVEC cells by promoting NO production and reducing the ROS and LDH released. Carnosol can upregulate *eNOS* gene expression and VE-cadherin protein expression. This occurs via ROS-mediating the activation of Nrf2-ARE pathway that appears to be responsible for inducing Nrf2 accumulation in the nucleus. It subsequently binds to ARE sequences and upregulates HO-1 and Nrf2 gene and protein expression. Our experiments only examined the effects of endothelial damage in HMVEC cells, and we need a vivo experiment of carnosol to improve the diabetic microangiopathy. In addition, we can conduct an intensive study of the potential mechanisms in the future.

## 4. Materials and Methods

### 4.1. Chemicals and Reagents

Carnosol was purchased from Solarbio (Beijing, China). Dulbecco’s modified eagle medium (DMEM) was purchased from Gibco (Grand Island, NY, USA). Fetal bovine serum (FBS) were purchased from Gibco BRL (Grand Island, NY, USA). Tert-Butyl hydroperoxide solution (t-BHP) was purchased from Macklin (Shanghai, China). PBS was obtained from Solarbio (PH 7.2–7.4) (Beijing, China). DCFH-DA was purchased from MedChemExpress (Monmouth Junction, NJ, USA). 

### 4.2. Cell Line and Culture

Human lung microvascular endothelial cells (HMVEC-L) and Human Embryonic Kidney Cells (HEK293) cell line was purchased from ATCC. Cells were maintained in DMEM supplemented with 10% FBS and 0.29 g/L L-glutamine, penicillin/streptomycin (1:100), in a humidified incubator at 37 °C and 5% CO_2_.

### 4.3. Antioxidant Activity by ABTS Method

Carnosol was dissolved in DMSO at a concentration of 40 mM for stock solution. Antioxidant activity detected by ABTS kit (Beyotime, Shanghai, China). The ABTS solution mixed with K_2_S_2_O_8_ to form a work solution and was stored at room temperature for 12 h–16 h before being used. Then the ABTS work solution was diluted with PBS for 40 times. 20 μL of carnosol at various concentration (10 ~ 0.04 μM) was mixed with 180 μL of diluted ABTS work solution. The absorbance at 734 nm was measured after incubation for 2–6 min at room temperature. We use the Absorbance (ODs) function of Flex station 3 (Molecular Devices, San Francisco, CA, USA) to detect the absorbance. 

### 4.4. CCK-8 Assay for Cell Viability Evaluation

HMVEC cells at a density of 9,000 per well were seeded in 96-well plates, incubated at 37 °C for 24 h, and treated with concentrations of carnosol for the 24 h, the cytotoxicity of carnosol was first assessed by the Cell Counting Kit-8 (CCK-8) assay (MCE, Monmouth Junction, NJ, USA). The cells were incubated with CCK-8 reagent (100 μL/mL medium) for 2 h, and the absorbance was read at 450 nm. Then they were treated with 200 μM t-BHP for 3 h. At the end of experiment, cell viability was evaluated by CCK-8 kit.

### 4.5. Determination of LDH

HMVEC were seeded in 96-well plates, incubated at 37 °C for 24 h, treated with carnosol for 24 h and then were treated with 200 μM t-BHP for 3 h. At the end of experiment, the supernatant for detecting the LDH release rate was sucked up with the LDH kits (Beyotime, Shanghai, China). This is a diaphorase-based INT color reaction. The LDH work solution was prepared according to the manufacturer’s protocols. The next steps involved pipetting 120 μL supernatant of each well which was added to the corresponding well of a new 96-well plate, then 60 μL LDH detection work solution was added to each well. The absorbance was read at 490 nm.

### 4.6. Annexin V-FITC/PI Double Staining

HMVEC cells were seeded in 96-well plates, incubated at 37 °C for 24 h, and pre-incubated carnosol for the 24 h and then were treated with 200 μM t-BHP for 3 h. After being washed with PBS, 100 μL 1× binding buffer was added, and was stained with 5 μL Annexin-V and 5 μL PI, then a fluorescence microscope was used to take photos. HMVEC cells at a density of 300,000 per well were seeded in 6-well plates, then treated with the same treatment for detection with a FACSC alibur flow cytometer (BD Sciences, San Jose, CA, USA).

### 4.7. Immunofluorescence

HMVEC cells (1.5 × 10^4^ cells/well) were seeded in a 96-well plate and were treated with 10 μM carnosol for 24 h. Then, the cells were treated with 200 μM t-BHP for 3 h and incubated with primary rabbit anti-VE Cadherin antibody (Abcam, Cambridge, UK) at 4 °C overnight. The cells were incubated with IgG H&L (Alexa Flour^®^ 488) for 1 h, and nuclei were covered with hoechest for 10 min. The fluorescence signals were imaged using fluorescence microscope (LEICA, Solms, Germany). We analyzed the fluorescence intensity of the line in Merged pictures via Image-Pro Plus.

### 4.8. Measurement of ROS and NO Generation

HMVEC cells were seeded in 6-well plates and treated with carnosol for the 24 h, then they were treated with 200 μM t-BHP for 3 h. The intracellular accumulation of ROS was detected using the DCFH-DA fluorescence assay. ROS generation was measured by the fluorescence intensity of dichlorofluorescein (DCF) at 525 nm after excitation at 488 nm using BD FACSC alibur flow cytometer. NO was detected by NO kit (Beyotime, Shanghai, China). The cells were loaded with DAF-FM DA (final concentration, 5 μM) for detecting the NO. Then we used a fluorescence microscope to take photos and analyzed the photos via Image J.

### 4.9. Molecular Docking Analysis

The crystal structure in complex with an inhibitor of Keap1 named 1VV (PDB code 4L7B, resolution 2.41 Å) was downloaded from the Protein Data Bank (www.rcsb.org). Excess protein side chain, incomplete residues, lack of hydrogen and the existence of waters molecules, were cleaned up by Discovery Studio 4.0 Client (DS 4.0). The active pocket was defined around 1VV. Then, carnosol was docked into the active pockets using CDOCKER methods.

### 4.10. Luciferase Reporter Activity Assay

To determine the ARE-agonistic activity of carnosol, we established a HEK293-ARE cell line. Luciferase Reporter Gene Assay Kit (Beyotime, Shanghai, China) was used to determine reporter gene activity in transiently transfected cells with FuGENE^®^ 6 Transfection Reagent (Promega, Madison, WI, USA). After transfection, cells at a density of 2.5 × 10^4^ per well were seeded in 96-well plates, incubated at 37 °C for 24 h, were treated with t-BHQ and carnosol (positive control) for 24 h. Cell lysates were then prepared for the assessment of luciferase activity.

### 4.11. mRNA Quantification by Real-Time PCR

HMVEC cells (3 × 10^5^ cells/well) were seeded in a 6-well plate and were treated with carnosol for 24 h and then were treated with 200 μM t-BHP for 3 h. Total RNA was isolated from HMVEC cells using TRIzol Reagent (Life Technologies, Carlsbad, CA, USA) according to the manufacturer’s protocol. After extracting total RNA from HMVEC cells, reverse transcription was performed prior to SYBR green real-time PCR analysis. Detection system is MA-6000 (Suzhou, China). The primers of objective genes and *GAPDH* primers were designed and shown in Table 1. 

### 4.12. Assessment Expression of HO-1 and Nrf2 Proteins

Antibodies for HO-1, β-actin were purchased from Proteintech (Rosemont, IL, USA), antibody for Nrf2 was purchased from Abcam (Cambridge, UK). HMVEC cells were seeded in a 6-well plate and were treated with carnosol for 24 h with different doses of carnosol or for the indicated time. The protein bands were detected by ECL reagents with the Bio-Rad ChemiDocTM MP Imaging System (Hercules, CA, USA). 

### 4.13. Statistical Analyses

Data is expressed as mean with standard deviation (SD). Analysis of variance (ANOVA) using Tukey’s test was applied to compare the mean of each group with that of the control group, and *p* value < 0.05 was considered to be statistically significant.

## Figures and Tables

**Table 1:** Primer sequences used for qRT-PCR analysis.

Gene	Sequence (5’–3’)	Product Length (bp)
*HO-1 F*	ACTGCGTTCCTGCTCAACAT	133
*HO-1 R*	GGGCAGAATCTTGCACTTTGT	
*Nrf2 F*	TCTGCCAACTACTCCCAGGT	124
*Nrf2 R*	ACGTAGCCGAAGAAACCTCA	
*eNOS F*	GCCGGAACAGCACAAGAGTTA	147
*eNOS R*	GCCCGAACACACAGAACCT	
*GAPDH F*	CTTTGTCAAGCTCATTTCCTGG	133
*GAPDH R*	TCTTCCTCTTGTGCTCTTGC	

## Abbreviations

HMVEC	human microvascular endothelial cells
LDH	lactate dehydrogenase
ROS	reactive oxygen species
NO	nitric oxide
VE-cadherin	vascular endothelial cadherin
Nrf2	nuclear factor (erythroid-derived 2)-like 2
Keap1	kelch-like ECH-associated protein 1
ARE	antioxidant-responsive element
t-BHP	tert-butyl hydroperoxide
ABTS	2,2’-azino-bis(3-ethylbenzothiazoline-6-sulphonic acid)
TBHQ	Tertiary butylhydroquinone
HO-1	Heme oxygenase 1
GSH	glutathione
GCLC	glutamate-cystein ligase catalytic
GST	glutathione-S-transferase
NQO1	NADP(H) quinone oxidoreductase 1
